# Antipyrin

**Published:** 1889-06

**Authors:** K. P. Moore

**Affiliations:** Macon, Ga.


### Southern Medical Record.

A MONTHLY JOURNAL OF PRACTICAL MEDICINE.

☞ All communications must be addressed to R. C. WORD, Managing Editor.

"*Quicquid Præcipies Esto Brevis.*"

VOL. XIX ATLANTA, GA., JUNE, 1889. NO. 6

### ORIGINAL AND SELECTED ARTICLES.

### ANTIPYRIN—ITS USE IN A NUMBER OF CASES.

From a paper read before the Medical Association of Georgia,

BY K. P. MOORE, M. D. OF MACON, GA.

In order to secure its antipyretic effect, I have not found it necessary to administer it in depressing doses. Of course, the larger the dose the greater the antipyresis. I have learned from experience and observation to be careful in its exhibition while the patient was well under the influence of quinia, for the antipyresis and depression is, I think, proportionately increased as our patient is more or less cinchonized. My own personal observation confirms me in the belief that it is indicated in any excess of pyrexia, whether due to traumatism, or idiopathic; whether to the puerperal state, or the result of a surgical operation.

The following cases have been selected with a view to their variety of causes, and to illustrate its usefulness in any case of fever, from whatever cause.

Case 1. Mr. R.; age about twenty; butcher; was taken with acute bilious fever. I gave calomel and rhubarb, followed by quinine, to complete cinchonism. The patient was kept thoroughly under the influence of quinia for forty-eight hours, with cold sponging, etc. He was very much nauseated, and constantly vomited bile. I saw him between eleven and twelve o'clock on the third day, with hot, dry skin, temperature 105½, and very sick. I ordered fifteen grs. antipyrine to be given every two hours until fever subsided. Saw him again at 6:30 p. m. Temperature 99; skin wet with perspiration; had had a delightful sleep, and, as he expressed it, he felt like a new man. Continued the antipyrine every five hours until the next day, when quinia was again resorted to, and the second day I dismissed the patient.

Case 2. Bessie S.; age eight months. Temperature 105; pulse 150; threatened with convulsions cause of fever, probably malarial. Ordered tepid bath, and antipyrine, four grs. every two hours. Saw her six hours later; fever all gone; little patient in fine perspiration, and comfortable. Thinking the fever might be due to acute indigestion, or some ephemeral cause, discontinued medicine and dismissed patient. Next day was called again; patient had had chill, and now had very high fever, antipyrine ordered as before, with like rapid and gratifying results. Quinine was now given, and patient made quick recovery.

Case 3. Maggie S.; age about twelve years. Found her in her second week of typhoid fever. Temperature, in afternoon, 104 to 104½. As is my custom, I gave her the benefit of the doubt, and instituted a course of quinia, aconite, gelsemium, etc. Failing with these measures to effect the fever, I ordered for her ten grs. of antipyrine every two or three hours. Unavoidable engagements prevented me from seeing her in the next twenty-four hours. Found her with temperature 97; cool surface; very weak pulse, and very much prostrated. Discontinued antipyrine. Applied heat and covering to surface and extremities, and gave whisky freely. She reacted slowly; and though for six weeks she lingered upon the threshold of the far-off beyond, yet, in God's good providence, she finally recovered. Did the depressing influence of the antipyrine, for the thirty-six hours while under its influence, play any part in the long drawn out fever? I think not, and although I did not again give her the antipyrine, yet I think now, after further experience with it, if I had the case to go over again I would do so, with more caution. My fright at the depression in which I found her, deterred me from trying it with her again.

Case 4. Mrs S.; age thirty; mother of four children. Was delivered in September, 1886, of a fine female child. Labor natural, and progress for several days satisfactory. On the third or fourth day she took a chill, followed in a few hours by temperature of 105¼, and pulse 146. I at once put her upon antipyrine, fifteen grs. every two and a half hours. In ten or twelve hours patient was in profuse perspiration; temperature 99½, and feeling quite comfortable. Hoping that this ill turn meant no serious trouble, I ordered a dose of oil and turpentine, and left the result with the future. Within the next thirty-six hours another chill, followed by high fever, was but the ominous breaking out of the smoldering flames; and the rapid swelling of the abdomen, with great tenderness in the pelvic region, gave unmistakable evidence of the serious mischief going on within. Antipyrine was again pressed until the temperature reached 100½. Quinia and opium, hot vaginal carbolized douches, turpentine stupes, etc., now come in as re-enforcements to the antipyrine, and for several days the warfare between a severe pelvic peritonitis and the best remedies at my command waged hot. The wonderful endurance of nature formed a sure foundation upon which we bridged her over, and though it was a close call, yet she recovered in due time.

What part did the antipyrine play in this case? I would not undertake to say that it influenced the final issue, or that it lessened the duration of the attack, but the fact remains, that as an antipyretic it did its part well, and contributed largely to the comfort of the patient. Does it not seem reasonable that, if it kept the pyrexia within due bounds, and by its now well-known analgesic properties made the patient more comfortable, her physical forces were thereby economized, her resources husbanded, and her case influenced for good in that proportion?

Within ten days from the confinement of this case, I had another case, within one block of her, who had the same trouble, went through the same history, with about the same treatment, and made a good recovery.

Case 5. Charlie B.; age twelve. Was first seen April 23d, 1887. Had been sick and listless for several days, and presented every indication of a continued fever. Following my habit of trying to break these fevers, I gave him colagogue cathartics, quinine, salicylates, aconite, gelsemium, etc. Several days were thus consumed. I then prescribed six grs. of antipyrine, every two hours until fever subsided, and directed the mother, who is a very intelligent lady, to discontinue, or prolong the intervals whenever the fever abated. He was thoroughly cinchonized when the antipyrine was commenced. In about eight hours after the beginning of the remedy, I was summoned in haste. I found him in a collapse ; surface cool ; profuse perspiration ; pulse slow and weak ; temperature 97. Gave hot whisky toddy, and used dry mustard friction. He soon reacted, and in two hours was sleeping happily. This is one of a number of cases in which I was satisfied of the fact that the antipyrine acted more powerfully after quinine to cinchonism. This boy had the usual turn of fifteen to twenty days fever, but got well. These cases selected with reference to their varying character, serve to illustrate the wide range for the use of this remedy as an antipyretic. Strictly as an antipyretic, I know of no remedy superior to it ; and I think it can sometimes be made to abort fevers of an inflammatory character, if seen in time.

One case comes to my mind just now, where the patient, a stout, robust man, had chill, followed by high fever, rapid breathing, rales in lung, cough with bloody expectoration, and every indication of pneumonia. I put him thoroughly under the influence of antipyrine, and at my visit the next day found him sitting up, breathing easy, very little cough, no fever, and needing my services no longer.

I can commend it as a safe and very certain antipyretic, and while we cannot yet say that it will modify the course of, or abort disease, it certainly does contribute to the comfort of those who are suffering from high temperature. If carelessly used it may do harm. We should avoid the depressing effect, and should only seek a physiological result ; and no effort should be made to produce a normal temperature in a disease of which pyrexia is a prominent characteristic. For instance, if we are treating a continued fever, with a temperature of from 103 to 105, we should not attempt to keep the fever lower than 99½ to 100 ; or in other words we should seek what might be termed its *physiological*, rather than its *toxical* effect.

AS AN ANALGESIC.

Antipyrine was first used and recommended only as an antipyretic. Later it was discovered that chinoline derivatives in general have more or less analgesic property, and of course antipyrine with the rest. As to whether it possesses this property in greater proportion than the others, is a question which seems as yet unsettled. From the literature upon the subject, it is probable that it does. Clinical investigation has shown that the analgesic property is not the same as that possessed by the known anodynes, but the rationale of its action is as yet ill-understood. And hence its physiological action on the central nervous system naturally invites our interest.

Coupe states that if antipyrine be given to animals, in doses of thirty to sixty grs., the subsequent administration of strychnine is unable to produce convulsions, while the samc done of strychnine, given alone, acts fatally. Again, if a dog is poisoned by strychnine, and death appears imminent, a hypodermic of thirty grs. of antipyrine suffices to check the convulsions and to restore normal respiration. Gley, while confirming these statements, asserts that an intravenous injection of two drs. of antipyrine causes in a dog a grave convulsive attack, or, in other words, acts like strychnine. Brown-Sequard also calls attention to this singular instance, that the drug, in small doses, acts antidotal to strychnine, while in large doses it produces a genuine intoxication of strychnine. (*The World's Med. Review.*) As an anodyne or analgesic to the various neuroses, this remedy seems destined to take high rank. As a remedy for acute rheumatism, nervous headache from any cause, and in many forms of neuralgia, it has been found to affect many remarkable cures. At a meeting of the Verein Fur Innere Medicine, at Berlin, October 18th, 1885, Dr. Franke read a paper upon antipyrine, especially in the treatment of acute rheumatism. Having prescribed it in thirty-four cases of this disease, he arrives at the conclusion, supported by the observations of Lenhartz and Alexander, that its action is that of a specific. Rapid recovery ensued on its administration in nine out of thirteen cases. He said it was superior to salicylic compounds—first, in the readiness of its administration, and second, in freedom from unpleasant physiological effects. He did not know of any contraindications to its employment, and summed up to the effect that antipyrine is an energetic specific anti-rheumatic agent.

Dr. John Blake White writes, that antipyrine, when administered in masterful doses, not only promptly relieves the symptoms of headache whenever present, whether resulting from disordered digestion, disturbance of the menstrual functions, loss of sleep, undue mental effort, or uræmia, but also possesses reliable prophylactic virtues against recurrent attacks of cranial neuralgia. Says he has been much impressed with the promptness of relief which often follows the administration of even a single dose of fifteen grs. The relief usually comes on within a half hour.—*Practitioner*, Nov., 1888.

I have come to look upon this as one of our most valuable remedies in all cases of hemicrania, and am daily becoming more and more impressed with its potency. For some weeks previous to February 25th, 1888, I was troubled with a facial neuralgia due to a diseased tooth. The paroxisms would be quite severe, now and then, with a constant nervous hyperesthesia of that side of my face. I had been keeping under the influence of quinine and gelsemium for some days, but never feeling entirely free from pain. On the afternoon of the above date I suffered unusually for several hours; at 5 p. m. I took my first dose of fifteen grs. of antipyrine, and in thirty minutes I was entirely free from pain, and feeling more refreshed and better every way than for several days. My pulse, when I took it, was 50; at the end of the half hour, and when I was free from pain, the pulse was 54. I had two paroxysms during the night following, and was promptly relieved each time, and went soundly to sleep, after taking fifteen grs. of antipyrine. On March 5th, I suffered for twenty-four hours with rheumatism in my shoulders and back of neck. As is the rule with our profession, I put off taking anything as long as possible, and endured the pain through the day, and until midnight. Finding that I could not sleep, I took fifteen grs. of antipyrine, and was soon wrapped in pleasant slumbers. I awoke next morning, at seven o'clock, to find my rheumatism gone; and up to date (March 9th) I have had no return of it. I have often used it in acute rheumatism, and have had larger percentages of relief, and with more promptness, from this remedy than from all others combined.

The remedy seems to be making for itself, in all neurotic troubles, quite a reputation, and clinical observations lead us to feel that it has come to stay.

It is well worth a trial, and, from my own personal observations, I can confidently commend it to your careful consideration. As before remarked, we should avoid giving it in toxical quantities. It is best to begin with small doses, and repeat if necessary every one to four hours until the desirable effect is obtained. The dose ranges from five to forty grs., and may be given in water, with some elegant syrup, or otherwise flavored to suit the taste; or it may be given in capsule, or compressed tablets. It has come to be a household remedy in my family, and my children are in the habit of resorting to it at once for the relief of headache, without reference to the cause, and almost invariably it gives relief in less than an hour.

This paper would be incomplete if I did not, before closing, drop a word of caution as to the incompatibility existing between antipyrine and the spirits of nitrous ether. Both possessing febrifuge properties, we are apt sometimes to combine them in our prescriptions. Antipyrine is said to possess basic properties, and forms salts with many acids. With the nitrous acid, it forms a crystalline, greenish, substance, which has received the name of isonitroso antipyrine, and which is very poisonous. Possessed as antipyrine is with these properties, and its compatibilities not yet being fully known, I am in the habit of exercising caution not to combine it with any of the acids.

